# Data on photo-nanofiller models for self-cleaning foul release coating of ship hulls

**DOI:** 10.1016/j.dib.2016.08.010

**Published:** 2016-08-09

**Authors:** Mohamed S. Selim, Sherif A. El-Safty, Maher A. El-Sockary, Ahmed I. Hashem, Ossama M. Abo Elenien, Ashraf M. EL-Saeed, Nesreen A. Fatthallah

**Affiliations:** a National Institute for Materials Science (NIMS), 1-2-1 Sengen, Tsukubashi, Ibaraki-ken 305-0047, Japan; b Petroleum Application Department, Egyptian Petroleum Research Institute (EPRI), Nasr City, 11727 Cairo, Egypt; c Graduate School for Advanced Science and Engineering, Waseda University, 3-4-1 Okubo, Shinjuku-ku, Tokyo 169-8555, Japan; d Chemistry Department, Faculty of Science, Ain Shams University, Cairo, Egypt; e Process Development Department, EPRI, Nasr City, 11727 Cairo, Egypt

**Keywords:** Nanofillers, Fouling release, Self-cleaning, Photo-bactericidal

## Abstract

The data presented in this article are related to the research article entitled “Smart photo-induced silicone/TiO_2_ nanocomposites with dominant [110] exposed surfaces for self-cleaning foul-release coatings of ship hulls” (Selimet al., 2016) [1]. This article reports on successfully designing and controlling TiO_2_ spherical single crystal photo-nanofillers and indicating evidence of fouling resistance after stimulation through UV radiation exposure. These data also reveal that the influence of well-dispersed spherical TiO_2_ nanoparticles (NPs) into the polymer matrix surface features on the prepared fouling release (FR) coating. Single crystal TiO_2_ nanospheres have played a large role in the scenario of photocatalysis due to its cost effectiveness, inert nature and photo stability. The model output and the surface and mechanical behavior data of the fabricated UV-irradiated silicone-based FR nanocoatings are made publicly available through analyzing nanocomposite topology, superhydrophilicity and self-cleaning efficiency in order to enable critical analysis of the tailored model. It also investigates the photo-bactericidal effect confirmed through biofilm coverage data disability. The modeled nanocomposites were subjected to comparable studies with other published models so as to understand how different UV-irradiated nano-scale parameters propagate and affect bulk film response.

**Specifications Table**

**Table:** 

Subject area	**Chemistry**
More specific subject area	**Nanomaterials sciences, marine Antifouling paints**
Type of data	**Table and figure**
How data was acquired	**XRD, Electron microscopy images, Static contact angle meter, survey**
Data format	**Analyzed**
Experimental factors	**Single crystal TiO**_**2**_**nanospheres were mixed with polydimethylsiloxane (PDMS) in various ratios and the nanocomposite films were cured via hydrosilation mechanism.**
Experimental features	**Intensive Characterization of the TiO**_**2**_**, nanocomposites surface wettability**
Data source location	**NIMS/EPRI (Japan, Egypt)**
Data accessibility	**Within this article**

**Value of the data**

• The huge potential of spherical single crystal TiO_2_ photocatalyst by reinforcing a composite material and effect of self-cleaning behavior are demonstrated in order to open up new possibilities in various fields.
• The data are useful for comparing purposes when addressing the influence of photocatalyst for reinforcing and environmental friendly antifouling.
• The importance of silicone/TiO_2_ nanocomposite manufacturing practice in final performance is demonstrated.
• The data are valuable for the nanomaterials synthesis and foul release coating design.

## 1 Data

Four figures and one table were provided to show the PDMS/spherical TiO_2_ nanocomposite characterization and investigation of data for applying as environmentally friendly marine antifouling paints. A schematic representation of fouling resistance mechanism of the UV-irradiated silicone nanocomposites is presented here.

## 2 Experimental design, materials and methods

**Scheme 1:**
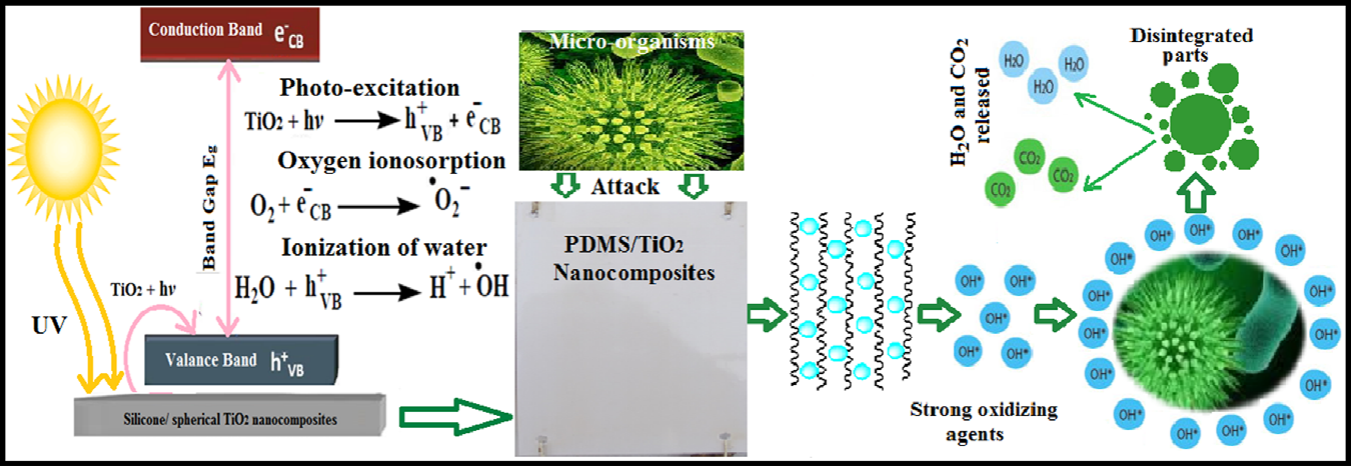
Mechanism of fouling resistance against biodegradation after UV irradiation for 0.5% nanofillers.

The data on the tailored nanocomposite surface and fouling resistance properties are obtained using various analytical techniques. The wetting characteristics of the prepared nanocomposites were studied through mathematical view point using proper mathematical tools. The reported mechanism of solar light boosted self-cleaning PDMS/spherical single crystal TiO_2_ nanocomposites mediated photocatalysis is illustrated in Scheme 1. Oxidation and reduction occur at the surface of TiO_2_ NPs, which change the wetting characteristics and improve superhydrophilic FR performance [2]. In this work, a comparable study indicated the higher performance of the tailored nanocomposites compared to other FR alternatives.

### 2.1 X-ray diffraction (XRD) measurements

**Fig. 1:**
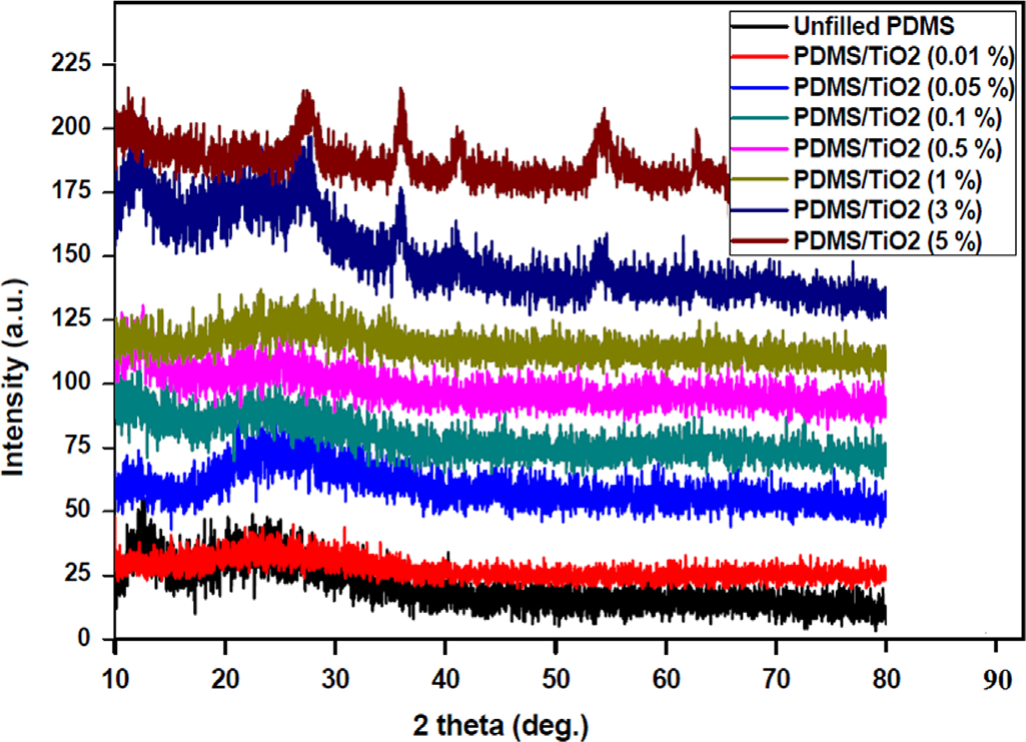
XRD spectra of PDMS/TiO_2_ nanocomposites with different nanofiller loadings.

XRD was used to characterize the layered structure of the polymer nanocomposites. The XRD patterns for the fabricated silicone/TiO_2_ nanocomposites were performed using a diffractometer (X׳Pert PRO model; PANalytical Corporation, Netherlands) with a monochromatic CuKα radiation source (*λ*=0.154) in 2*θ* scan range from 10° to 90°. The XRD results were illustrated in Fig. 1. Our finding indicates that the PDMS/TiO_2_ nanocomposites possess rather broad diffraction peaks, suggesting a small degree of crystallinity and a successful incorporation of rutile TiO_2_. At low TiO_2_ NP concentrations used, XRD plot shows no distinct peak, which approves a complete dispersion of the NPs inside the PDMS matrix.

### 2.2 Polarized light microscopy (PLM)

**Fig. 2:**
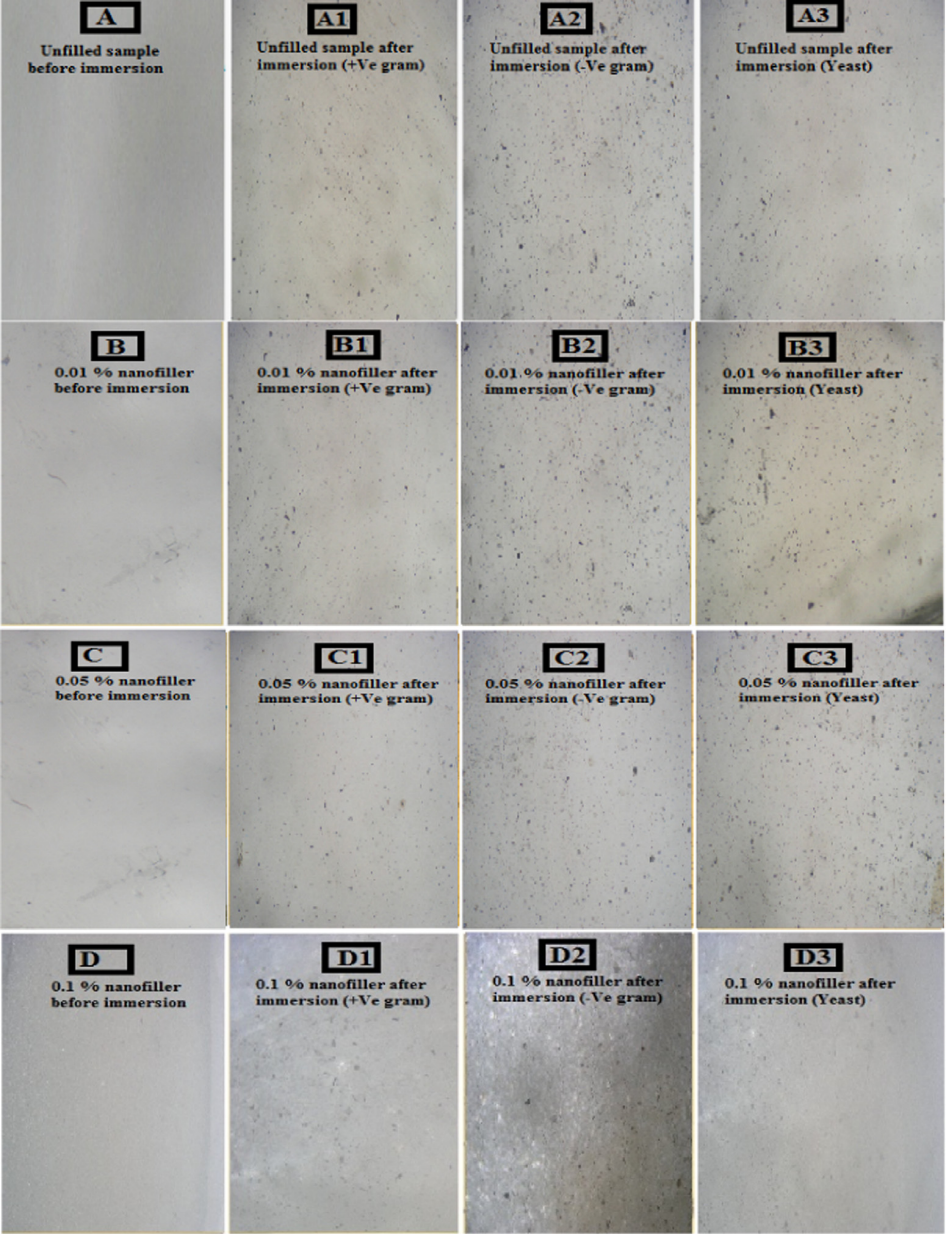

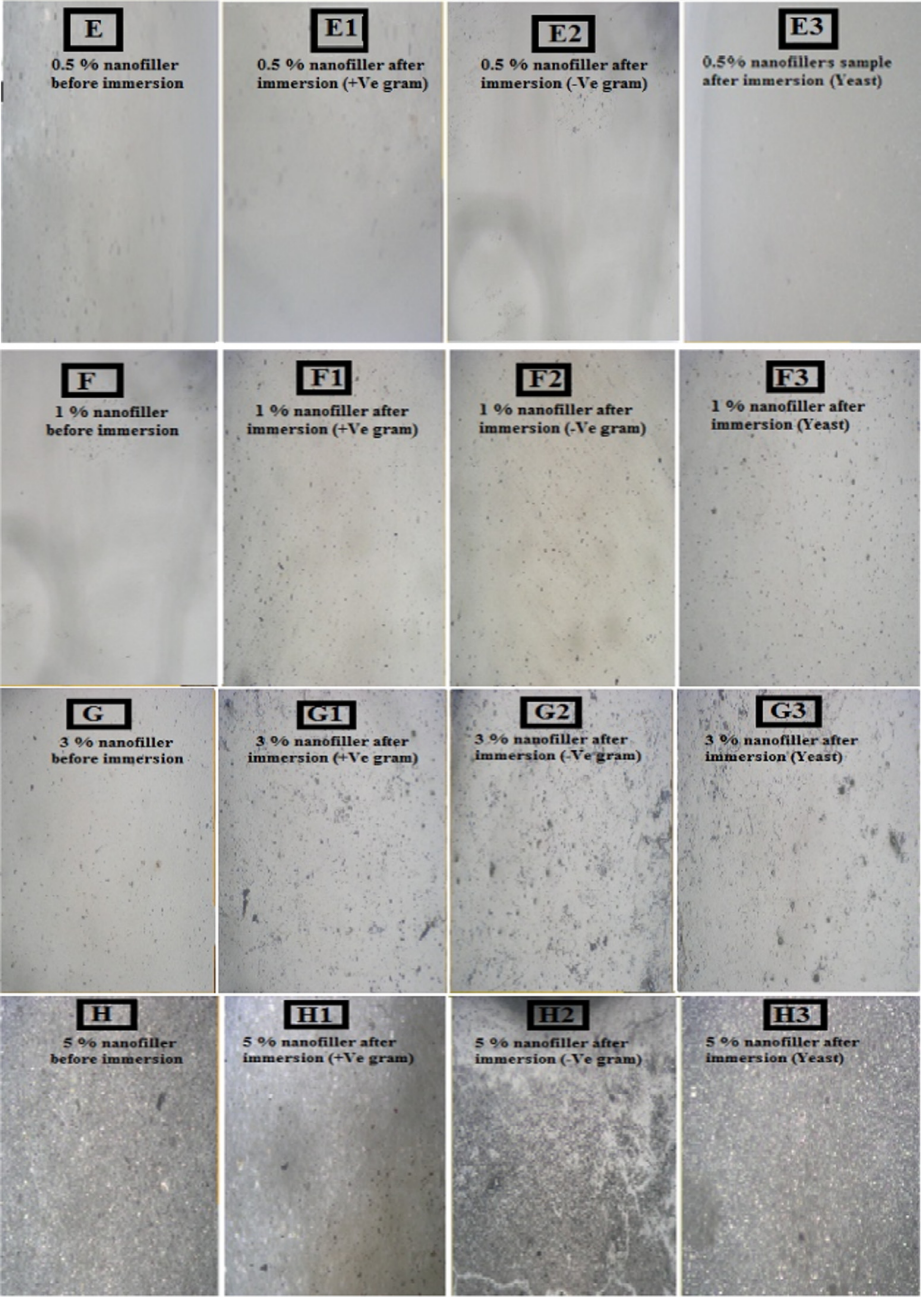
Optical microscope images (A), (A1), (A2) and (A3) of the unfilled PDMS; (B), (B1), (B2) and (B3) of the 0.01% nanofillers in the PDMS/TiO_2_ nanocomposites; (C), (C1), (C2) and (C3) of the 0.05% nanofillers in the PDMS/TiO_2_ nanocomposites; (D), (D1), (D2) and (D3) of the 0.1% nanofillers in the PDMS/TiO_2_ nanocomposites; (E), (E1), (E2) and (E3) of the 0.5% nanofillers in the PDMS/TiO_2_ nanocomposites; (F), (F1), (F2) and (F2) of the 1% nanofillers in the PDMS/TiO_2_ nanocomposites; (G), (G1), (G2) and (G3) of the 3% nanofillers in the PDMS/TiO_2_ nanocomposites; (H), (H1) (H2) and (H3) of the 5% nanofillers in the PDMS/TiO_2_ nanocomposites; all before and after immersion in bacterial strains for 1 month.

PLM is used to determine the biofilm coverage of the utilized microorganisms on the surface of unfilled and filled PDMS/TiO_2_ nanocomposites (Fig. 2.). PLM micrographs of the specimens were captured using Olympus BH-2 microscope (Japan) and by using Image J software program. The homogeneity of the surface observed after immersion in the microorganism. The surface immunity against fouling is observed at low concentration (up to 0.5%) due to the well-dispersion of TiO_2_ nanospheres inside the PDMS matrix. On contrarily, nonhomogeneity of the surface is observed for the unfilled samples due to the fouling settlement and with increasing nanofiller loadings of up to 5%. The specimens were also densely fouled because agglomeration that may break down the failure mechanism for the adhesion of fouling organisms.

### 2.3 Scanning electron microscopy (SEM)

**Fig. 3:**
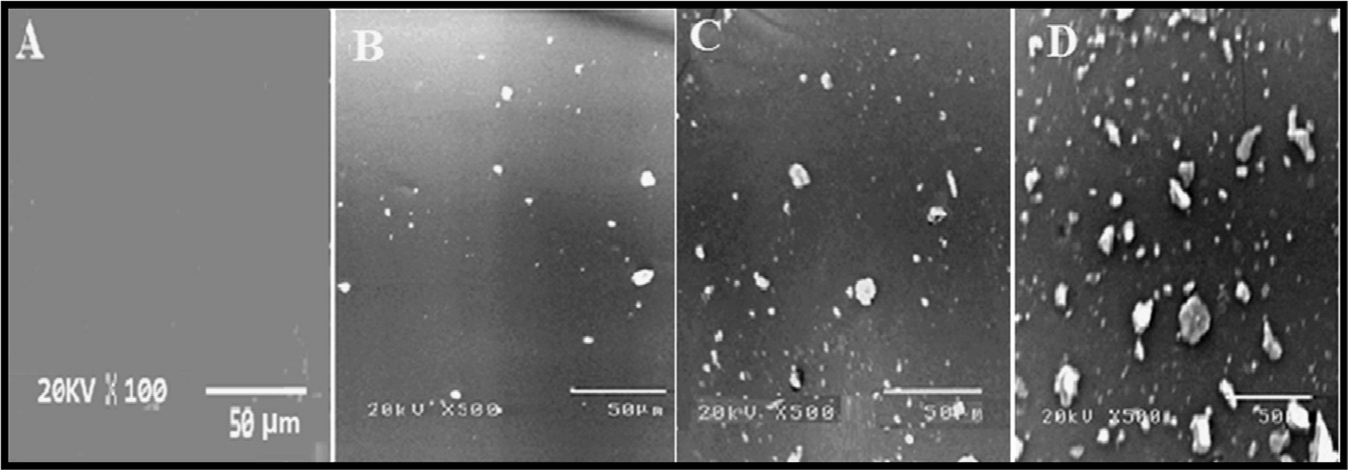
SEM images of the field test sample (A) before immersion, (B), (C) and (D) are SEM images at different positions of the panel after 1 year of immersion.

SEM images were captured using a JSM530 (JEOL, Japan) instrument with an accelerating voltage of 20 keV. SEM micrographs were carried out on the fabricated silicone enriched TiO_2_ (0.05%) nanocomposites as shown in Fig. 3 before and after immersion for 1 year. The homogeneity of the surface is observed before immersion. On contrarily, after 1 year of immersion SEM images indicated the settlement of fouling microorganisms on the surface and thus heterogeneity was observed without sever colonization, thus affording enhanced immunity against micro-organisms.

### 2.4 Mechanical tests of the unfilled and filled PDMS

In order to evaluate the mechanical properties of the prepared unfilled and silicone/TiO_2_ nanocomposites (0.5%), three different tests, namely, impact, cross cut and T-bending were performed. The tests reflect the elasticity, adhesion strength and flexibility of the modeled coatings. A 17 cm×9 cm×1 mm steel panel was degreased and coated with an epoxy resin primer coat. A second coat of epoxy/silicon (50:50) was applied as a tie coat. Then, a final coat of the PDMS/TiO_2_ nanocomposite coating was applied with a dry film thickness of 150 µm. Impact tester (Ref BG5546, Sheen Instruments. Ltd., UK) was used to perform impact tests according to ISO 6272 to determine the height of sudden falling load (1 kg weight) on the coated PDMS and resistance to damage of the prepared films. Results of the impact test show that no cracks were observed, indicating the high elasticity and flexibility of the tested nanocomposite coatings (Table 1). The adhesion degree of the prepared films was tested by a cross-cut test using a sharp steel cutter (Sheen 750, UK), and the grid size was 2 mm×2 mm. The adhesive strength was examined by using adhesion tape which rated according to ASTM D 3359. The cross cut test was carried out without resulting visible adhesion defects (Table 1). The T-bending test was carried out on the coated specimens (Ref 809 type model, Sheen Instruments Ltd., Kingston, UK) through ISO 6272. The measurements were based on the spindle diameter. No intrusion was identified under a magnifying glass in any of the tested paints after penetration and bending on a <5 mm cylindrical spindle (Table 1).

## 3 Surface measurements and comparable study

This work demonstrates the design, fabrication and field exposure study of novel series of PDMS/spherical rutile TiO_2_ nanocomposites. We synthesized and applied vinyl terminated PDMS owing to its superior properties as compared to hydroxyl terminated analogues in our previous study [3], [4], [5]. Rutile single crystal TiO_2_ photo-nanospheres were synthesized, controlled successfully, dispersed in the PDMS matrix with different proportions from 0.01% up to 5% and cured using hydrosilation curing pathway. The different concentrations were analyzed before and after UV illumination.

**Fig. 4:**
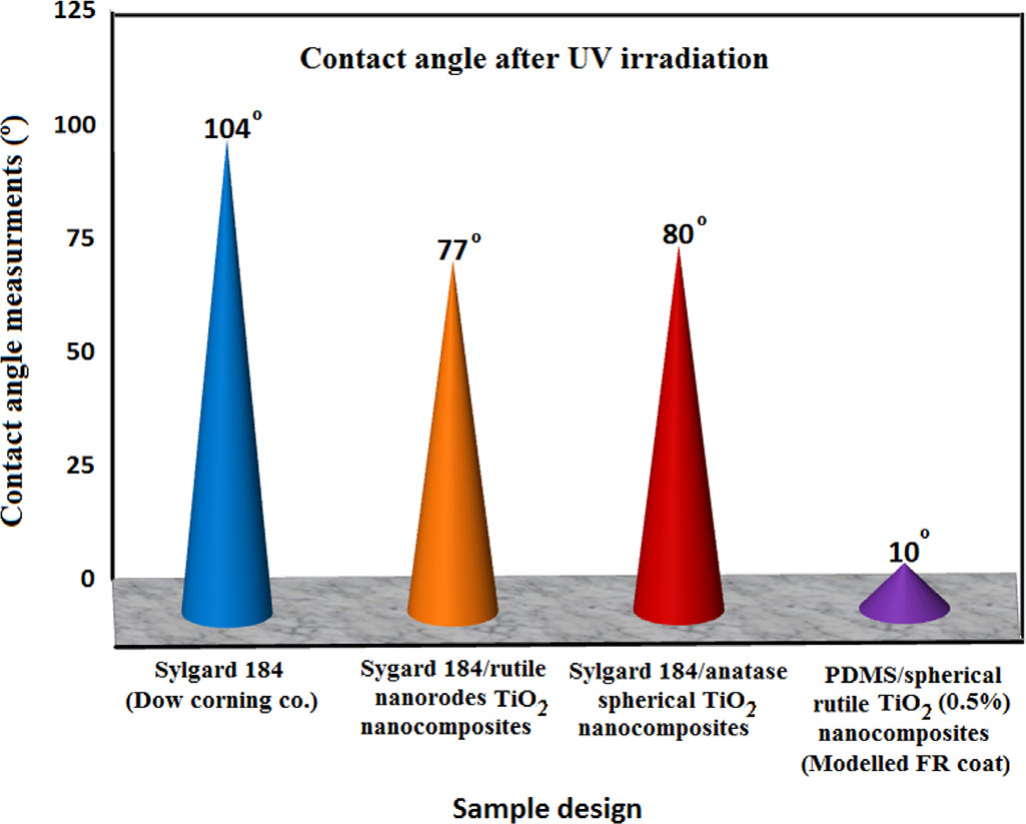
Comparison of the prepared PDMS/TiO_2_ nanocomposites and other commercial FR coatings.

By comparing the static contact angle measurements for Sylgard^®^184 silicone film as a control sample [6], PDMS/nanorods TiO_2_ composites [6], PDMS/anatase spherical TiO_2_ nanocomposite [6] and our newly designed PDMS/spherical single crystal rutile TiO_2_ nanocomposite with its superior concentration (0.5% TiO_2_ NPs), the superior FR performance was observed for out tailored nanocomposites. The comparison based on contact angel change after pulsed by UV irradiation as shown in Fig. 4, where the contact angle remains unchanged after UV irradiation for unfilled Sylgard®184, decreased to 77° for PDMS/nanorods TiO_2_ composites and to early 80° for PDMS/anatase spherical TiO_2_ nanocomposite [7]. On the other hand, our developed model (0.5%) decreased the contact angle to 10° after UV irradiation indicated superhydrophilicity and increased self-cleaning ability and thus higher AF performance. These results were confirmed by Δ*G*_sl_ which exerted superhydrophilicity (−144.49 mJ/m^2^) and inhibit fouling cohesion on the ship hull surface. Also these results were confirmed by biological assays and field exposure tests which approved the improved durability and self-fouling-releasing performance. Many excellent characteristics of single crystal rutile TiO_2_ (110 crystal plane) have promoted its application in photocatalysis as being chemically inert, high light scattering, and inexpensive. The protection mechanism against biodegradation was discussed in Scheme 1.

## Figures and Tables

**Table 1:** Mechanical tests of unfilled and (0.5%) spherical single crystal TiO_2_ filled nanocomposite.

**Sample design**	**Impact test (*J*)**	**Crosshatch test**	**Bending test (mm)**
PDMS blank	5	Pass	<5
PDMS/TiO_2_ nanocomposite (0.5%)	10	Pass	<5
